# A rare case of pericardial sarcoidosis presenting as chest pain

**DOI:** 10.1002/ccr3.9160

**Published:** 2024-07-14

**Authors:** Husam El Sharu, Prarthana Jain, Bart Singer, E. Amanda Snyder

**Affiliations:** ^1^ Department of Internal Medicine East Carolina University Brody School of Medicine Greenville North Carolina USA; ^2^ Division of Rheumatology, Allergy & Immunology, Department of Medicine University of North Carolina Chapel Hill North Carolina USA; ^3^ Department of Pathology and Laboratory Medicine University of North Carolina Chapel Hill North Carolina USA

**Keywords:** cardiac sarcoidosis, chest pain, pericardial sarcoidosis, sarcoidosis

## Abstract

**Key Clinical Message:**

Pericardial sarcoidosis is an uncommon cause of chest pain to consider, and it requires a heightened level of suspicion and thorough history gathering. If there is suspicion of inflammatory disease, pursuing advanced imaging and biopsies is crucial, as early immunosuppressive treatment can enhance outcomes.

**Abstract:**

Pericardial involvement in sarcoidosis is a rare condition with limited research. This case study discusses a 52‐year‐old African American woman who presented with subacute chest pain and was diagnosed with pericardial sarcoidosis. Diagnostic evaluation revealed extensive lymphadenopathy and pericardial effusion, and a pericardial biopsy confirmed non‐caseating granulomatous inflammation. Treatment with steroids and methotrexate resulted in clinical improvement. Eight months follow‐up showed near resolution of pericardial disease. This case emphasizes the importance of considering cardiac sarcoidosis in sarcoidosis patients, utilizing advanced imaging for accurate diagnosis, and tailoring treatment to the level of cardiac involvement.

## 1 INTRODUCTION

Sarcoidosis is a systemic inflammatory disease characterized by non‐caseating granulomas in the affected organs. In most cases, sarcoidosis chronically progresses to involve the lungs and the hilar lymph nodes. Other extrapulmonary manifestations include the skin, eyes, nervous system, and heart.^1^ Cardiac Sarcoidosis (CS) frequently remains asymptomatic in patients with pulmonary or systemic sarcoidosis, as evidenced by autopsy studies revealing a 25% prevalence of silent CS among these individuals. However, only 5% of sarcoidosis patients experience symptomatic cardiac manifestations.^2^ Clinical presentation of CS is variable, ranging from silent electrocardiographic abnormalities to arrhythmias such as heart block, ventricular tachycardias leading to sudden cardiac death, or nonischemic cardiomyopathy leading to heart failure. CS has a propensity to involve the myocardium, specifically the interventricular septum.^2^

The diagnosis of CS is complex and likely underdiagnosed. It requires clinical suspicion and integrating clinical and pathologic information with results of advanced cardiac imaging, such as cardiac magnetic resonance imaging (CMR) or 18F‐fluorodeoxyglucose‐positron emission tomography (FDG‐PET).^3^ It is worth noting that cardiac symptoms can serve as the initial presentation leading to the diagnosis of sarcoidosis.^2^

Pericardial involvement is rare and usually associated with myocardial infiltration and may be found in 12% of patients with CS.^4^, ^5^ Isolated pericardial involvement is rarely reported and is challenging to diagnose due to its variable presentation. It can range from asymptomatic pericardial effusion to acute or subacute pericarditis or, in rare cases, pericardial tamponade, all of which can be attributed to a variety of other differential diagnoses.^5^ Herein, we report a case of a 52‐year‐old female, who presented to the hospital with chest pain and was found to have isolated pericardial sarcoidosis.

## 2 CASE PRESENTATION

A 52‐year‐old African American female presented to the emergency department with subacute onset left‐sided chest pain for the past 7 months associated with progressive shortness of breath. A review of systems (ROS) was concerning for 50 lb. weight loss, fatigue, and neck swelling. Medical history was significant for essential hypertension, but she denied having significant past surgical, family, or social history. The physical examination showed no abnormalities, with regular heart rhythm, lungs clear to auscultation, absence of friction rubs, no signs of lower extremity edema, and no rashes.

**FIGURE 1:**
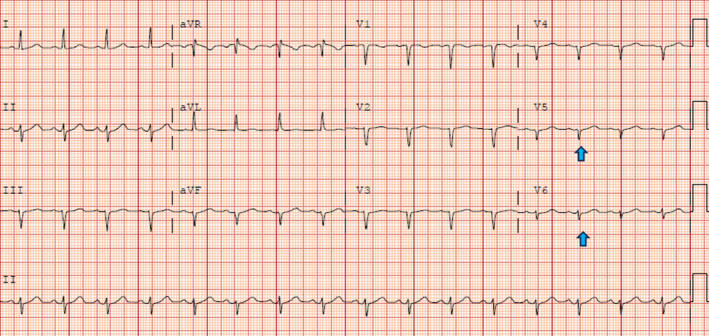
The patient's EKG shows left axis deviation and low voltage QRS (blue arrows) without evidence of AV block (PR interval of 152).

**FIGURE 2:**
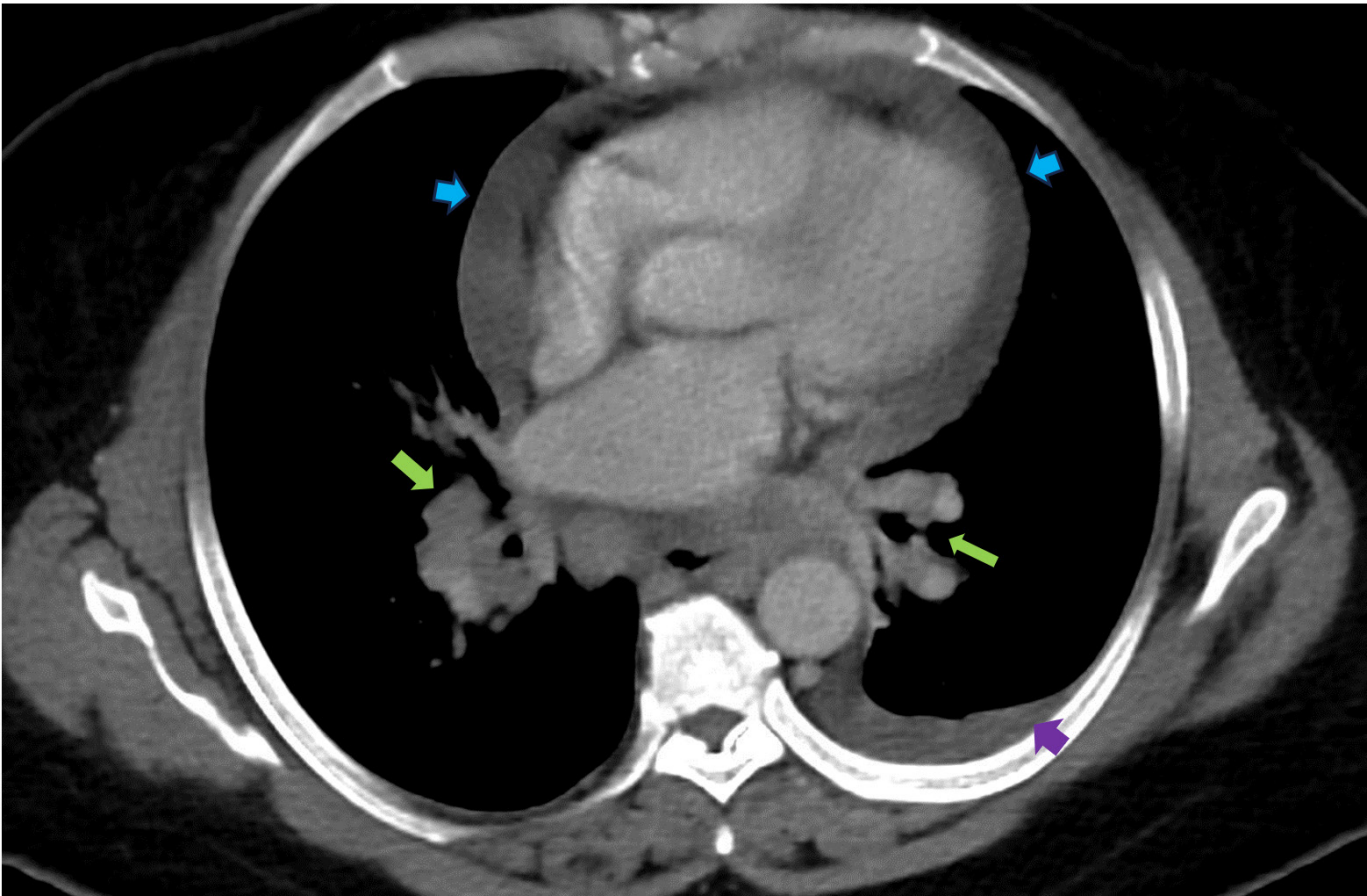
CTA of the chest: Blue arrows indicate a moderate‐size pericardial effusion. The purple arrows highlight the left‐sided pleural effusion. The green arrows show evidence of bulky hilar lymphadenopathy bilaterally.

Initial labs, including complete blood count, metabolic panel, and troponins, were unremarkable. She had an angiotensin‐converting enzyme level of 150 U/L and a soluble form of the interleukin‐2 receptor of 1451 pg/mL. Chest x‐ray (CXR) showed bilateral perihilar opacities without evidence of pulmonary infiltrates. Multiple electrocardiographies (EKGs) demonstrated left axis deviation and low voltage QRS complexes but no conduction abnormalities, including atrioventricular (AV) block (Figure 1). Computed Tomography Angiography (CTA) was performed to rule out pulmonary embolism (PE) and did not show evidence of pulmonary artery defects. However, she was incidentally found to have diffuse lymphadenopathy, including supraclavicular, mediastinal, bihilar, and paratracheal nodes, along with a moderate‐sized pericardial effusion and a small left pleural effusion (Figure 2).

## 3 DIFFERENTIAL DIAGNOSIS AND WORKUP

**FIGURE 3:**
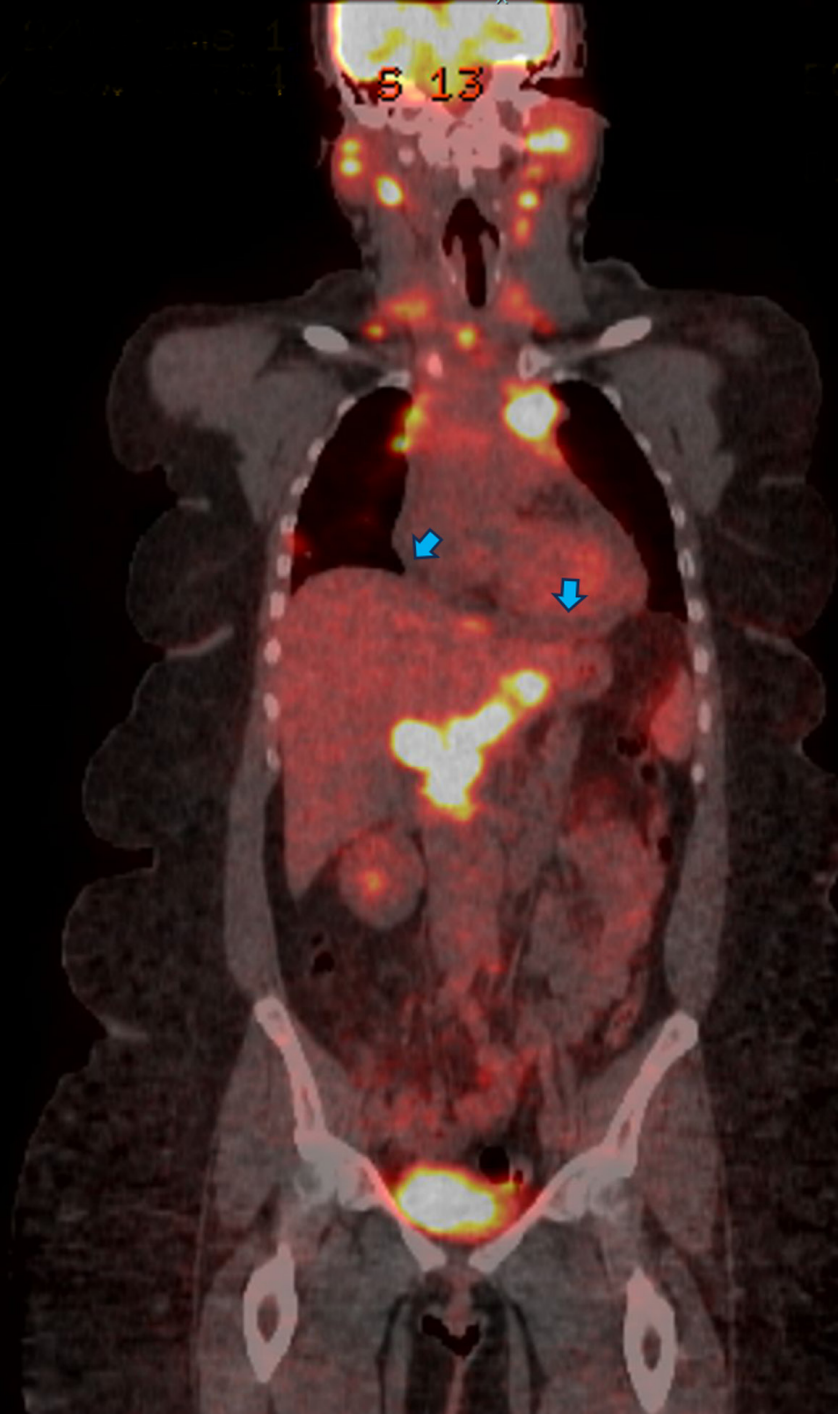
A coronal view of the whole body FDG‐PET fusion scan shows evidence of diffuse lymphadenopathy. The blue arrows indicate a small pericardial effusion with no active metabolization activity.

The differential diagnosis of chest pain in a patient with sarcoidosis includes regular differential diagnoses such as cardiovascular, pulmonary, or musculoskeletal causes of chest pain. She had a CTA of the chest, which suggested the diagnosis of lymphoma, given the presence of diffuse lymphadenopathy. Therefore, a fluorodeoxyglucose (FDG)‐positron emission tomography (PET) was obtained and showed extensive hypermetabolic adenopathy in the neck, chest, abdomen, and pelvis, a hypermetabolic subpleural lung nodule, and small pericardial effusion (Figure 3).

**FIGURE 4:**
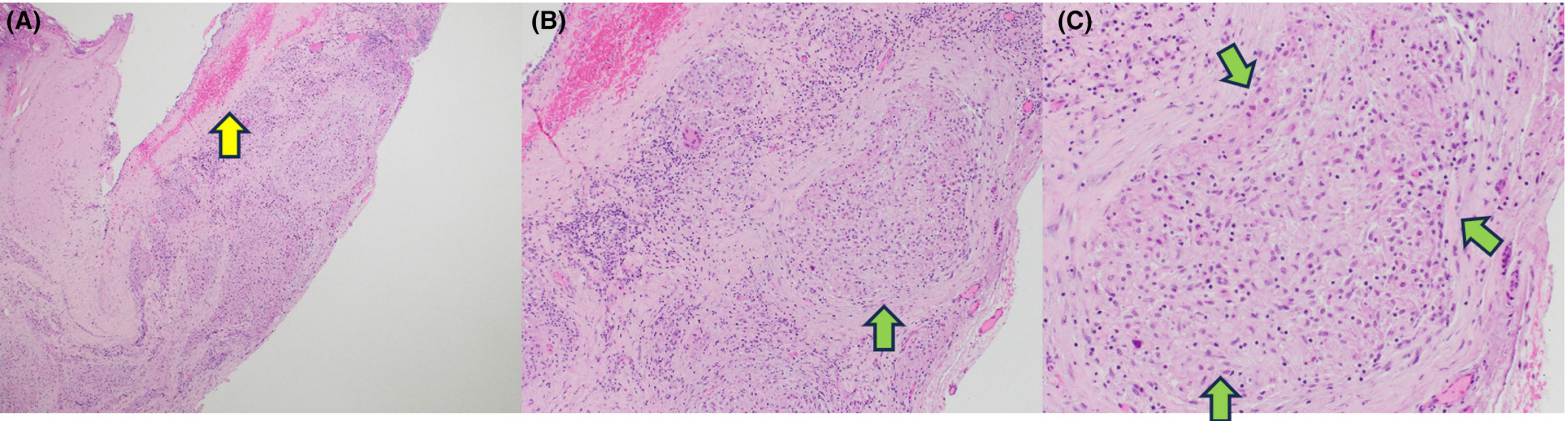
A hematoxylin and eosin‐stained section of the pericardium. Figure 1A (4×) shows fibrous pericardial tissue with a mesothelial layer (Yellow arrow) and a central stromal expansion by nodular, noncaseating granulomas, best seen in Figure 1B, C (10× and 20×) (Green arrows).

A percutaneous biopsy of her right supraclavicular lymph node showed non‐caseating granulomas with negative stains for mycobacteria and fungi, including acid‐fast bacteria and Grocott methenamine silver stains. Flow cytometry showed no definitive immunophenotypic evidence of lymphoproliferative disorder. Due to her extensive lymphadenopathy (with consideration for malignancy), she underwent video‐assisted thoracoscopic surgery (VATS), pericardial window, and pleurodesis. Cytology of the pericardial fluid showed reactive mesothelial cells, macrophages, and lymphocytes with no identified malignant cells. Additionally, the patient underwent an excisional pericardial biopsy, which revealed fibrous pericardial tissue with extensive non‐necrotizing granulomatous inflammation, with negative staining for mycobacteria and fungi and no evidence of malignancy (Figure 4). A myocardial biopsy was not obtained due to low suspicion of myocarditis.

## 4 OUTCOMES AND FOLLOW‐UP

**FIGURE 5:**
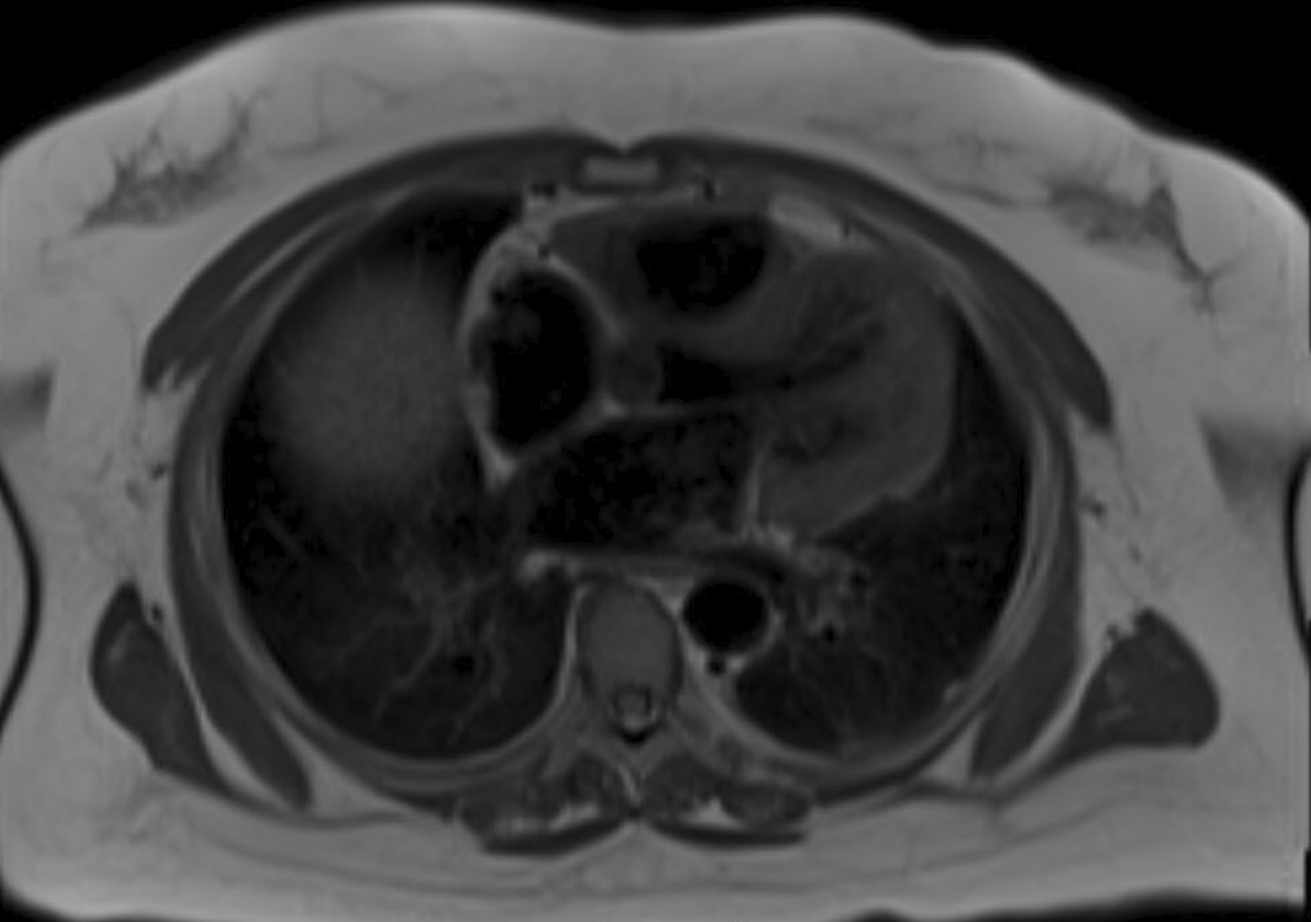
T1‐weighted CMR 2 months after treatment shows the absence of myocardial scarring or fibrosis. Additionally, there is no thickening or enhancement of the pericardium.

The patient was started on prednisone 0.5 mg/kg/d. Given her weight of 120 kg, she received a total daily dose of 60 mg/d. After 2 months with insufficient symptom control, she began taking oral methotrexate 15 mg weekly while tapering off prednisone. A subsequent CMR revealed no myocardial scarring, fibrosis, or late gadolinium enhancement of the myocardium. A trace pericardial fluid was noted on CMR, but there was no thickening or enhancement of the pericardium. (Figure 5).

**FIGURE 6:**
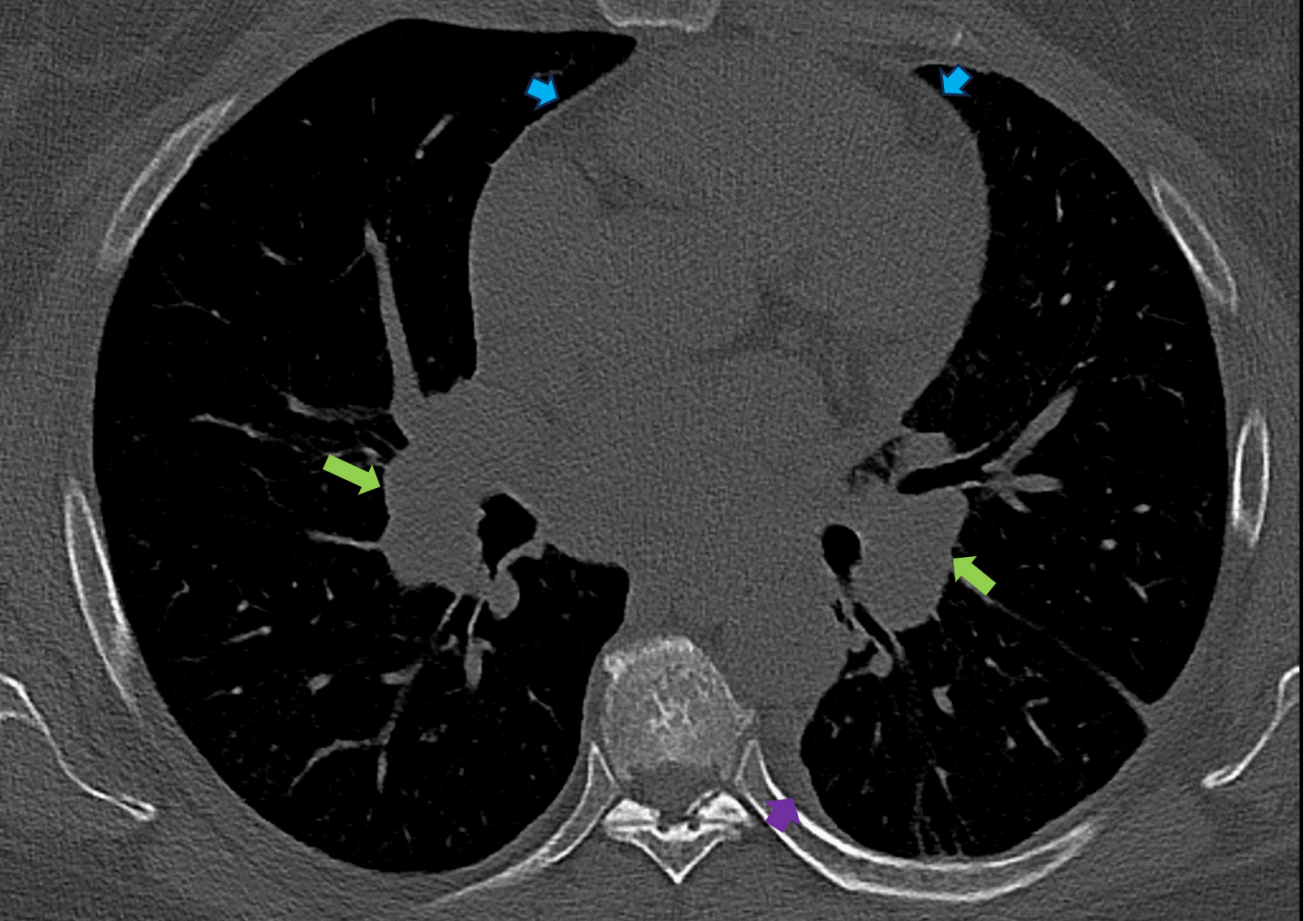
CT chest 8 months after treatment. The blue arrows indicate significant interval improvement in the pericardial effusion. The purple arrows highlight the near resolution of the left‐sided pleural effusion. The green arrows show the persistence of bulky hilar lymphadenopathy bilaterally.

Six months later, her chest pain improved, and a cardiac PET scan indicated no active cardiac inflammation, though extracardiac sarcoidosis in the spleen and mediastinum were still present. An echocardiogram showed an ejection fraction of 55%, normal left ventricle size and diastolic function, and a resolution of pericardial effusion (Videos 1 and 2). A follow‐up chest CT was obtained 8 months after treatment, showing near resolution of her pericardial effusion with the persistence of lymphadenopathy (Figure 6).

**VIDEO 1:** This is an apical four‐chamber view of the patient's echocardiography 6 months after starting treatment. It shows the normal size and thickness of the ventricles without evidence of diastolic dysfunction.

**VIDEO 2:** This is a parasternal long‐axis view of the patient's echocardiography 6 months after starting treatment. The echocardiogram shows an ejection fraction of 55% and a resolution of pericardial effusion.

## 5 DISCUSSION

Sarcoidosis is an inflammatory disease manifested by non‐caseating granulomas that can present in any organ, including the heart.^1^ CS has a broad spectrum of presentations depending on the location and extent of the disease, the most concerning being sudden cardiac death due to arrhythmias from the involvement of the conduction system in the myocardium.^3^

Pericardial sarcoidosis is rare and usually results from the spread of myocardial infiltration.^6^ It typically manifests as a small pericardial effusion, reported in 20% of CS cases. The pericardial effusion is typically transudative, noninflammatory, and straw‐colored but could be serosanguinous. A meta‐analysis of pericardial sarcoid cases by Mahalwar et al. revealed that almost 90% of patients had pericardial effusion, and 48% had constrictive pericarditis and tamponade.^5^ The time course can be variable, ranging from acute pericarditis to, more commonly, insidious onset pericarditis evolving over months.^7^

The most common symptom in patients with pericardial sarcoidosis is dyspnea related to pericardial effusion. Like our patient, they may also experience significant chest pain. However, unlike our patient, pulmonary, and myocardial involvement is often present in those with pericardial disease, with incidence rates of 77% and 59%, respectively.^5^ Although not necessary for diagnosis, our case is distinctive as only a few cases of pericardial sarcoidosis have been definitively diagnosed through a biopsy directly from the pericardium.

Diagnosing CS clinically is challenging. Biopsy, specifically endomyocardial biopsies, is the gold standard for diagnosing CS, with high specificity and prognostic value, as myocardial involvement correlates with shorter survival. A myocardial biopsy can also rule out other diseases, such as giant cell myocarditis and eosinophilic myocarditis, which can present similarly to sarcoidosis.^8^ However, endomyocardial biopsy has limited sensitivity due to the patchy nature of the disease, especially when the disease is solely in the myocardium.^9^ Uemura et al. found that only 20% of endomyocardial biopsies in patients with known sarcoidosis and highly suspected cardiac involvement detected non‐caseating granulomas. As a result, treatment was administered to patients with high suspicion of cardiac involvement, even if their biopsies were negative.^10^ Currently, there is no consensus on the role of the biopsy for patients with isolated pericardial involvement.

Diagnosis methods encompass electrocardiography and echocardiography, although these exhibit low sensitivity and specificity. Thallium 201 and technetium 99 m scintigraphy offer intermediate levels of sensitivity and specificity.^8^ With advancing imaging modalities, FDG‐PET, and CMR had the highest sensitivity and specificity for diagnosing CS, even in patients without clinical or electrical evidence of disease.^9^ According to Japanese Ministry of Health and Welfare guidelines, Smedema et al. found CMR's sensitivity to be 100% and specificity to be 78% in CS patients.^11^

Due to the variety of presentations, CS can have different treatment options. Specific cardiac treatments depend on the extent of the disease, with options ranging from antiarrhythmics to implantable cardioverter‐defibrillators and, in some patients, cardiac transplantation.^4^ In patients with pericardial sarcoidosis, a pericardial window might be required if effusions persist despite immunosuppressive therapy.^5^, ^7^

Like other types of sarcoidosis, CS usually requires immunosuppressive treatment. The indication to start treatment includes any symptomatic disease or unequivocal symptoms with evidence of inflammation on PET or CMR.^12^ Birnie et al. suggested that CS patients should be initially treated with Prednisone 0.5 mg/kg/day for 2–3 months, followed by a PET scan to assess response. If the disease is still active, adding a steroid‐sparing agent should be considered.^2^ Although the efficacy of corticosteroids and appropriate dosing were not assessed in large, randomized prospective studies, various retrospective studies supported their use.^8^

Steroid‐sparing agents, including methotrexate, azathioprine, mycophenolate mofetil, and azathioprine, have been used. Other biologics, including anti‐tumor necrosis factor inhibitors, such as infliximab or adalimumab, have also been used with good efficacy. However, reported outcomes on each agent for treating pericardial sarcoidosis are sparse.^12^ Our patient achieved a good response with methotrexate. The specific regimen is chosen based on experience, patient preference, and co‐morbidities. Studies are limited, however, regarding sufficient data to support the use of one agent over another.^4^ Additionally, achieving a balance between minimizing steroid‐related side effects and the risk of infection with immunosuppression is also vital.

In some patients with pericardial sarcoidosis, colchicine, and nonsteroidal anti‐inflammatory drugs have also been used, especially in those presenting with symptoms of pericarditis.^5^

## 6 CONCLUSION

This case highlights the importance of considering pericardial involvement in sarcoidosis, especially in patients with bihilar opacities, lymphadenopathy, and pericardial effusion. Advanced imaging and timely biopsy are crucial for accurate diagnosis. Effective treatment with steroids and methotrexate can lead to significant clinical improvement. This case underscores the need for heightened clinical awareness and a multidisciplinary approach in managing this underdiagnosed aspect of cardiac sarcoidosis.

## AUTHOR CONTRIBUTIONS

**Husam El Sharu:** Conceptualization; methodology; project administration; writing – original draft; writing – review and editing. **Prarthana Jain:** Supervision; validation; visualization; writing – review and editing. **Bart Singer:** Visualization; writing – review and editing. **E. Amanda Snyder:** Supervision; validation; writing – review and editing.

## FUNDING INFORMATION

None.

## CONFLICT OF INTEREST STATEMENT

The authors have no conflict of interest to declare.

## CONSENT

Written informed consent was obtained from the patient to publish this report in accordance with the journal's patient consent policy.

## Data Availability

All generated and analyzed data for this study are included in the manuscript.

## References

[1] Bokhari SRA , Zulfiqar H , Mansur A . Sarcoidosis. StatPearls [Internet]; 2023. Accessed November 5, 2023 https://www.ncbi.nlm.nih.gov/books/NBK430687/

[2] Birnie DH , Nery PB , Ha AC , Beanlands RSB . Cardiac Sarcoidosis. J Am Coll Cardiol. 2016;68(4):411‐421. 27443438 10.1016/j.jacc.2016.03.605

[3] Katsadouros V , Vincent D . A case of isolated cardiac sarcoidosis: an underdiagnosed disease with little diagnostic consensus. An Intern Med Clin Cases. 2022;1(10):e220762. Accessed November 5, 2023. doi:10.7326/aimcc.2022.0762.

[4] Nunes H , Freynet O , Naggara N , et al. Cardiac sarcoidosis. Semin Respir Crit Care Med. 2010;31(4):428‐441. Accesses November 5, 2023 https://pubmed.ncbi.nlm.nih.gov/20665393/ 20665393 10.1055/s-0030-1262211

[5] Mahalwar G , Kumar A , Agrawal A , et al. Pericardial Involvement in Sarcoidosis. Am J Cardiol. 2022;170:100‐104. 35227500 10.1016/j.amjcard.2022.01.025

[6] Ipek E , Demirelli S , Ermis E , Inci S . Sarcoidosis and the heart: a review of the literature. Intractable Rare Dis Res. 2015;4(4):170‐180. Accessed November 5, 2023 https://pubmed.ncbi.nlm.nih.gov/26668777/ 26668777 10.5582/irdr.2015.01023 PMC4660858

[7] Kinney E , Murthy R , Ascunce G , Donohoe R , Zelis R . Pericardial effusions in sarcoidosis. Chest. 1979;76(4):476‐478. Accessed November 5, 2023 https://pubmed.ncbi.nlm.nih.gov/477439/ 477439 10.1378/chest.76.4.476

[8] Kim JS , Judson MA , Donnino R , et al. Cardiac sarcoidosis. Am Heart J. 2009;157(1):9‐21. Jan. [Accessed November 5, 2023] https://pubmed.ncbi.nlm.nih.gov/19081391/. 19081391 10.1016/j.ahj.2008.09.009

[9] Doughan AR , Williams BR . Cardiac sarcoidosis. Heart. 2006;92(2):282‐288. Feb. [Accessed November 5, 2023] https://pubmed.ncbi.nlm.nih.gov/16415205/ 16415205 10.1136/hrt.2005.080481 PMC1860791

[10] Uemura A , Morimoto SI , Hiramitsu S , Kato Y , Ito T , Hishida H . Histologic diagnostic rate of cardiac sarcoidosis: evaluation of endomyocardial biopsies. Am Heart J. 1999;138(2 Pt 1):299‐302. 10426842 10.1016/s0002-8703(99)70115-8

[11] Smedema JP , Snoep G , Van Kroonenburgh MPG , et al. Evaluation of the accuracy of gadolinium‐enhanced cardiovascular magnetic resonance in the diagnosis of cardiac sarcoidosis. J Am Coll Cardiol. 2005;45(10):1683‐1690. May 17. [Accessed November 5, 2023] https://pubmed.ncbi.nlm.nih.gov/15893188/ 15893188 10.1016/j.jacc.2005.01.047

[12] Lehtonen J , Uusitalo V , Pöyhönen P , Mäyränpää MI , Kupari M . Cardiac sarcoidosis: phenotypes, diagnosis, treatment, and prognosis. Eur Heart J. 2023;44(17):1495‐1510. May 1. [Accessed November 5, 2023]. doi:10.1093/eurheartj/ehad067 36924191 PMC10149532

